# Non-Contact Blood Pressure Prediction Using Radar with a Lightweight Temporal Multi-Scale Feature Fusion Network

**DOI:** 10.3390/s26113468

**Published:** 2026-05-31

**Authors:** Yuhan Liu, Tianlin Zhang, Yonggang Luo, Liguo Zhou, Li Ding, Yinwei Li

**Affiliations:** 1 School of Optoelectronic Information and Computer Engineering, University of Shanghai for Science and Technology, Shanghai 200093, China; 2335050905@st.usst.edu.cn (Y.L.); 243350630@st.usst.edu.cn (T.Z.); 2 Henan Medical College, Zhengzhou University, Zhengzhou 450001, China; luoyg_514@126.com; 3 Terahertz Spectrum and Imaging Technology, Co-Operative Innovation Center, Terahertz Technology Innovation Research Institute, University of Shanghai for Science and Technology, Shanghai 200093, China; zlg@usst.edu.cn (L.Z.); sunnylding@usst.edu.cn (L.D.); 4 Nantong Taiji Technology Co., Ltd., Nantong 226152, China; 5 Shanghai Institute of Intelligent Science and Technology, Tongji University, Shanghai 200092, China; 6 School of Intelligent Emergency Management, University of Shanghai for Science and Technology, Shanghai 200093, China

**Keywords:** hypertension, non-contact measurement, radar, neural network, blood pressure prediction

## Abstract

**Highlights:**

**What are the main findings?**

- To address the issue of network model complexity in existing millimeter-wave radar blood pressure prediction methods, a lightweight temporal multi-scale feature fusion network, LULMNet, is proposed. This network maintains low model complexity while preserving both blood pressure prediction accuracy and waveform reconstruction fidelity.
- An explicit multi-scale feature fusion strategy for mid-to-deep layers is incorporated into the model to address the limitation of existing methods, which primarily rely on a single deep feature during the blood pressure regression stage.

**What are the implications of the main findings?**

- Establishing an accurate and regular blood pressure monitoring system can facilitate the early detection of hypertension risks and the scientific prevention of cardiovascular and cerebrovascular complications.
- The network designed in this paper performs particularly well in the small error range, further enhancing the prediction accuracy and stability of the model in both blood pressure prediction and waveform reconstruction.

**Abstract:**

Hypertension is a major global health issue, and continuous, convenient blood pressure monitoring is of great significance for early screening and intervention. To address the insufficient exploitation of multi-scale temporal features and the high model complexity in existing radar-based non-contact blood pressure prediction methods, we propose a lightweight temporal multi-scale feature fusion network (LULMNet), for blood pressure prediction and waveform reconstruction. LULMNet adopts a two-stage training strategy. In the first stage, a lightweight one-dimensional U-Net (1D U-Net) is employed for blood pressure waveform reconstruction and intermediate-to-deep temporal feature extraction. In the second stage, systolic and diastolic blood pressure are estimated via multi-scale fusion of intermediate and deep features from the encoder of the 1D U-Net, followed by LSTM-based temporal modeling and regression through a global average pooling (GAP)and a two-layer fully connected prediction head. Experimental results show that the proposed model achieves an error of 3.21 ± 4.94 mmHg for systolic blood pressure (SBP) and 2.25 ± 3.39 mmHg for diastolic blood pressure (DBP), satisfying the Grade A standard of the British Hypertension Society (BHS). In addition, the normalized mean absolute error (NMAE) for waveform reconstruction is as low as 0.044. These results indicate that the proposed method maintains low model complexity while ensuring prediction accuracy, with only 3.0 M parameters and 0.37 G floating-point operations (FLOPs), demonstrating strong potential for non-contact continuous blood pressure monitoring.

## 1. Introduction

In its Global Report on Hypertension published in 2025, the World Health Organization (WHO) noted that approximately 1.4 billion people aged 30–79 were living with hypertension worldwide in 2024, but only about one-fifth had achieved effective blood pressure control through medication or lifestyle interventions [1]. Hypertension can lead to a range of complications, including heart disease [2], stroke [3], and kidney failure [4]. It may severely impair the function of vital organs such as the heart, brain, and kidneys, and can ultimately result in disability or death. In addition, hypertension can also have adverse effects on mental health, and patients with this condition generally face a higher risk of comorbid depression and anxiety [5]. Continuous monitoring of blood pressure changes before the onset of clinical symptoms can provide early warning of abnormal upward trends in blood pressure, thereby helping prevent the development of hypertension. Therefore, establishing an accurate and routine blood pressure monitoring system is an effective approach for the early identification of hypertension risk and the scientific prevention of cardiovascular and cerebrovascular complications.

At present, blood pressure is measured predominantly using traditional inflatable cuff devices. Inflatable cuff-based blood pressure measurement typically relies on the oscillometer method, where cuff pressure changes induce periodic brachial artery volume variations with each heartbeat, generating pressure oscillations [6]. Systolic blood pressure and diastolic (SBP and DBP) can then be estimated from the characteristic relationship between these pressure oscillations and cuff pressure. However, cuff compression and measurement-related anxiety may activate the sympathetic nervous system, leading to a transient elevation in blood pressure, commonly known as “white coat hypertension” [7]. In addition, some individuals may be allergic to the cuff material, making it impossible to complete the measurement [8]. In contrast, contactless measurement can collect data with little or no awareness by the subject, making it possible to obtain blood pressure readings under natural and relaxed conditions. Such data are more representative of the subject’s true blood pressure status and have greater clinical reference value.

At present, the main research directions in contactless blood pressure measurement include imaging photoplethysmography (rPPG) [9,10], thermal imaging [11], and radar techniques [12]. rPPG enables noninvasive and remote measurement of volumetric pulse wave signals by using multi-wavelength cameras to detect subtle pulse-induced color changes on the skin surface [13]. Because of relying on optical absorption, its accuracy is susceptible to interference from ambient light and is further affected by variations in skin tone and translucency. Thermal imaging uses infrared cameras to capture subtle skin temperature fluctuations in facial and other regions caused by cardiac pulsation, thereby extracting pulse-related temperature variation signals [14]. However, because infrared radiation has limited penetration capability, the target area must remain unobstructed by clothing, bedding, or other coverings during measurement. In addition, facial scanning may raise concerns about privacy leakage. Radar techniques transmit electromagnetic signals toward the human body and capture variations in the reflected signals caused by subtle arterial motions induced by cardiac activity, thereby extracting pulse wave information [15]. By detecting physical displacement through electromagnetic waves, radar-based methods are less affected by the limitations of optical sensing and therefore offer more stable measurement performance. And they only output waveform data and do not collect any visual image information. As a result, they offer better environmental robustness, stronger penetration capability, and greater privacy protection. These advantages make them suitable for a wider range of applications.

In recent years, research on radar-based noninvasive continuous blood pressure prediction has advanced steadily, with extensive efforts devoted to key issues such as feature extraction, blood pressure prediction, and waveform reconstruction. With the development of computing technology, pulse wave data processing methods have gradually evolved from early physics-based modeling to machine learning, and further toward deep learning approaches represented by neural networks. As a core tool of deep learning, neural networks can perform efficient feature extraction through multilayer nonlinear transformations [16], enabling them to capture deep blood-pressure-related features from radar-measured pulse wave signals. Owing to this advantage, neural networks have become an important technical approach in current research on radar-based blood pressure prediction.

Existing studies suggest that deep learning methods have considerable potential to improve blood pressure prediction accuracy, but several limitations remain in practical applications. Liu et al. proposed the mmRBP system based on a deep transfer learning framework and demonstrated the feasibility of noninvasive continuous blood pressure monitoring using a single frequency-modulated continuous-wave (FMCW) millimeter-wave radar [17]. However, the system requires highly controlled experimental conditions: subjects must remain seated still for 60 s and keep the wrist fixed in an upward position. These constraints limit its convenience for daily use and reduce its adaptability to real-world measurement scenarios. Wang et al. proposed a two-stage deep learning network that integrates ResNet and Transformer, achieving certain improvements in noise suppression and spatiotemporal feature extraction [18]. However, the introduction of multi-head self-attention and cross-stage feature fusion leads to high computational cost and increased inference latency. Wang et al. proposed a temporal-spatial feature fusion network (TSFN) that combines ResNet, gated recurrent units, and a multi-head attention mechanism [19]. Although this model improves blood pressure prediction performance, its cascaded multi-module architecture likewise increases the computational burden, making it less suitable for deployment in low-computing-power scenarios. Qiu et al. proposed RSD-Net, which improves estimation accuracy by enlarging the feature extraction window [20]. While this strategy expands the range of temporal information utilized, it also increases the data processing burden and prediction latency, thereby limiting the model’s real-time applicability. In addition, Jiang et al. embedded a self-attention mechanism into the multi-resolution modules of MultiResUNet and combined CNN with LSTM for blood pressure prediction [21]. Although this model achieves relatively high prediction accuracy, the increase in network parameters and the incorporation of attention modules substantially raise model complexity and inference cost. Moreover, the intermediate multi-scale features in MultiResUNet are not fully exploited. Therefore, how to maintain or even further improve blood pressure prediction accuracy while minimizing model complexity and hardware dependence remains an important issue that deserves further investigation in this field.

To address the difficulty of balancing prediction accuracy and model complexity in existing methods, this study proposes a deep learning model, termed LULMNet, that takes both aspects into account. The model adopts a lightweight one-dimensional U-Net (1D U-Net) as its backbone and incorporates an LSTM with multi-scale feature fusion together with global average pooling (GAP). This design enables it to capture multidimensional temporal features in physiological signals as well as the intrinsic dependencies among these features. Based on these key features, the model can accurately predict SBP and DBP, as well as high-fidelity blood pressure waveform reconstruction.

The main contributions of this study are summarized as follows:

(1) To address the issue of network model complexity in existing millimeter-wave radar blood pressure prediction methods, a lightweight temporal multi-scale feature fusion network, LULMNet, is proposed. This network maintains low model complexity while preserving both blood pressure prediction accuracy and waveform reconstruction fidelity.
(2) An explicit multi-scale feature fusion strategy for mid-to-deep layers is incorporated into the model to address the limitation of existing methods, which primarily rely on a single deep feature during the blood pressure regression stage.
(3) Ablation experiments were designed and conducted to systematically analyze the contribution of different key modules to the blood pressure prediction performance. The results validated the effectiveness of the proposed model and its components, providing evidence for the rationale of the model design.

Section 2 introduces the methods for blood pressure prediction and waveform reconstruction. Section 3 describes the training environment and training strategy. Section 4 presents the experimental results. Section 5 provides a discussion, including ablation and comparative experiments. Finally, the main conclusions of the study are summarized.

## 2. Methodology

### 2.1. Data Preprocessing

The raw I/Q echo signals acquired by the millimeter-wave radar are jointly affected by multiple factors, including subtle body surface vibrations induced by cardiac activity, respiratory motion, static clutter, and system noise. As a result, these signals typically exhibit pronounced amplitude fluctuations, baseline drift, and multi-source component aliasing, making them unsuitable for direct pulse waveform extraction and subsequent model training. To obtain high-quality signals that can faithfully characterize cardiac pulsation features, it is necessary to preprocess the raw echoes. The main objectives of preprocessing are as follows:

(1) to enhance the effective micro-motion components caused by cardiac activity;
(2) to suppress interference from respiratory harmonics, background clutter, and random noise;
(3) to improve the signal-to-noise ratio and waveform stability.

**Figure 1:**
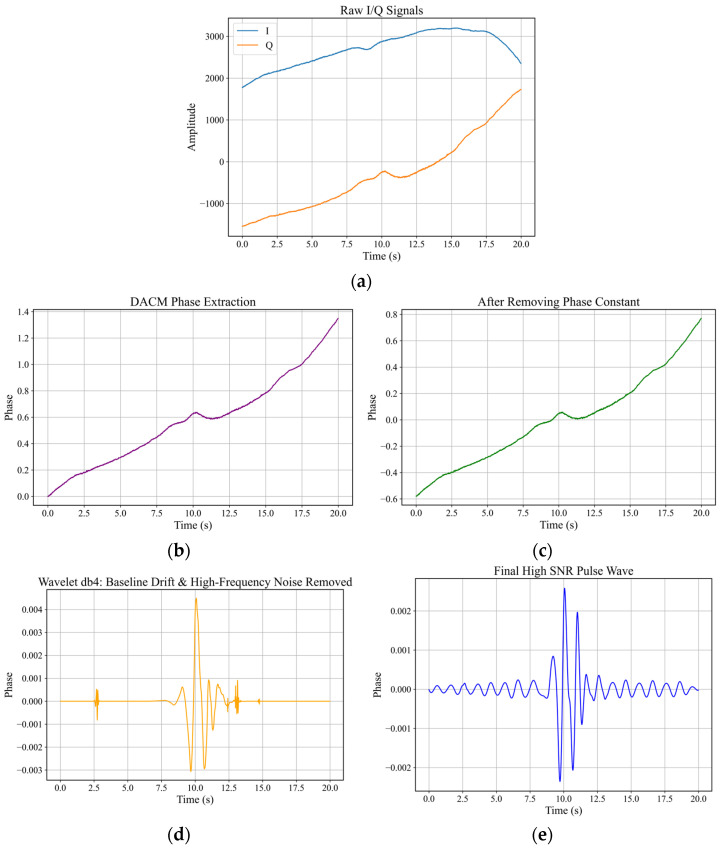
Pulse wave extraction process (**a**) raw I/Q signals; (**b**) phase signal extracted by DACM; (**c**) phase signal after removal of the phase constant; (**d**) signal after wavelet denoising and baseline drift correction; (**e**) final processed signal.

After preprocessing, the usability of the radar echoes can be effectively improved, thereby providing reliable, high-quality, and feature-distinct input data for the subsequent training and validation of the neural network. The following preprocessing pipeline was established [21]:

Before signal preprocessing, abnormal segments were first excluded according to data quality criteria. Segments with obvious motion artifacts, saturated or abrupt changes in the radar I/Q signals, missing or abnormal BP waveforms, or physiologically implausible BP values were removed. The remaining valid continuous signals were then used for the subsequent preprocessing procedure shown in Figure 1.

Figure 1 illustrates the signal processing procedure. Figure 1a shows the raw I/Q dual-channel echo signals acquired, where the amplitudes of the I and Q signals exhibit a slow variation over time, containing subtle fluctuations induced by body-surface micro-motion. First, the differential cross-multiplication algorithm (DACM) is applied to extract phase information from the raw I/Q signals [22]. Specifically, the instantaneous phase variation was estimated from the differential changes in the I and Q channels and then accumulated to obtain the continuous phase sequence. As shown in Figure 1b, the phase waveform exhibits regular fluctuations, including fast rhythmic micro-motions from cardiac activity and slow rhythmic variations from respiration. Compared with direct analysis of amplitude signals, the phase sequence can more sensitively reflect subtle body surface displacements caused by physiological motion.

Static reflectors and environmental clutter introduce phase constants that cause an overall shift in the micro-motion signal. Applying global mean filtering to the extracted phase sequence yields a baseline with symmetric fluctuations around zero. In the implementation, this step was performed by subtracting the mean value of the extracted phase sequence from the original phase signal. As shown in Figure 1c, the peaks and troughs are more balanced relative to the baseline. The baseline of the phase sequence is corrected to near zero, and the overall trend remains consistent. However, the waveform is now centered around zero, with fluctuations distributed symmetrically around it, making the previously masked subtle fluctuations easier to observe.

After mean-centering, the constant phase offset in the phase signal is removed. However, slowly varying low-frequency baseline drift and high-frequency random noise may still remain. To further improve the quality of the radar-derived pulse wave signal, db4 wavelet decomposition is employed in this study to denoise the continuous phase signal and correct baseline drift. The db4 wavelet has compact support and favorable time-localization properties, enabling effective noise suppression while preserving transient morphological features of the pulse wave, such as local peaks, valleys, and rapid upstrokes. Based on these properties, db4 was selected as a suitable wavelet basis for denoising radar-derived pulse wave signals, because it provides a practical balance between noise suppression, baseline drift correction, and preservation of short-duration local morphological variations in the time domain [23]. In the experiments conducted in this study, the maximum decomposition level of the db4 wavelet was set to 7. Considering that the practically achievable decomposition level is jointly constrained by the length of the continuous signal and the length of the db4 filter, the final decomposition level is defined as follows:

(1) $$L=min(7,L_{max})$$

here, $L_{max}$ denotes the maximum decomposition level permitted for the current continuous phase signal when using the db4 wavelet.

After wavelet decomposition, the lowest-frequency approximation coefficients are set to zero to remove the slowly varying baseline drift in the phase signal. For the high-frequency detail coefficients at each decomposition level, the noise standard deviation is estimated using the median absolute deviation (MAD):

(2) $$\sigma_{j}=\frac{median(\left|c_{j}\right|)}{0.6745}$$

here, $c_{j}$ denotes the wavelet detail coefficients at the j-th decomposition level. Subsequently, the threshold for the detail coefficients at the j-th decomposition level is calculated according to the universal thresholding criterion:

(3) $$\lambda_{j}=\sigma_{j}\sqrt{2\ln N_{j}}$$

here, $N_{j}$ denotes the length of the detail coefficients at the j-th decomposition level. Based on this threshold, soft-threshold shrinkage is applied to the detail coefficients at each decomposition level to attenuate high-frequency random noise. Finally, the denoised phase signal is reconstructed using the inverse discrete wavelet transform. As shown in Figure 1d, the overall slow upward trend present in the signal before processing has been completely eliminated, and the signal baseline is stabilized near zero, effectively suppressing baseline drift. Meanwhile, the originally smooth phase trend in the waveform is transformed into significant pulse-like fluctuations concentrated around 10–12 s, while the signal in other time periods remains near zero, indicating that random noise has been effectively filtered out.

Finally, to further isolate respiratory interference and enhance the cardiac components, a notch filter is applied to suppress respiration and its lower-order harmonics. This notch filtering strategy was designed to suppress respiratory components and their harmonics. The dominant respiratory frequency was first estimated from the wavelet-denoised phase signal within 0.1–0.5 Hz. Then, IIR notch filters were applied at the estimated respiratory fundamental frequency and its first three harmonics, with the quality factor Q set to 30. After respiratory harmonic suppression, a fourth-order Butterworth band-pass filter with a passband of 0.8–4.0 Hz was applied to extract the cardiac-related pulse component. Zero-phase filtering was used to avoid phase distortion during filtering. As shown in Figure 1e, the cardiac-related periodic pulse features become dominant, with no apparent low-frequency drift or high-frequency noise interference. The pulse wave signal is clearly periodic, with distinct peaks and troughs, providing a reliable basis for high signal-to-noise ratio pulse extraction.

### 2.2. Construction of the LULMNet

#### 2.2.1. LULMNet Overview

**Figure 2:**
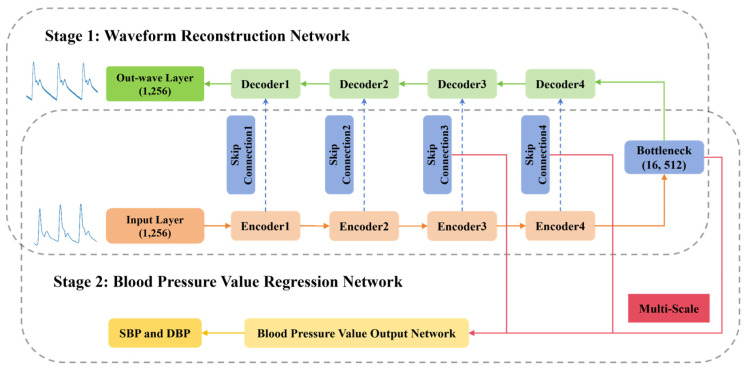
Architecture of the LULMNet.

This study proposes a lightweight temporal multi-scale feature fusion network, termed LULMNet, as shown in Figure 2. The overall model consists of two main modules: a lightweight four-layer one-dimensional U-Net backbone and a blood pressure value output network. The 1D U-Net is responsible for signal encoding, feature extraction, and waveform reconstruction. The blood pressure regression network consists of the encoder from the 1D U-Net and a blood pressure output network, which together enable the precise prediction of blood pressure values.

#### 2.2.2. Backbone 1D U-Net

The model backbone adopts a 1D U-Net with a four-layer encoder–decoder structure. The U-Net architecture is commonly used for 2D image segmentation [24]. However, its symmetric encoder–decoder structure and core design of skip connections can be effectively adapted to handle one-dimensional temporal waveform signals. In this study, the 2D convolutions in the encoder–decoder convolutional blocks are replaced with 1D convolutions, each with a kernel size of 3 and a stride of 1, making the network suitable for processing one-dimensional temporal blood pressure waveforms. Downsampling in the encoder is performed using 1D max-pooling with a pool size of 2 and stride of 2. Let $C_{in}$ denote the number of input channels, $C_{out}$ denote the number of output channels, and k denote the convolution kernel size. The number of parameters of the two convolution operations can then be expressed as follows:

(4) $${Params}_{1D}=C_{in}\cdot C_{out}\cdot k$$

(5) $${Params}_{2D}=C_{in}\cdot C_{out}\cdot k^{2}$$

When k=3 is used in this study, the parameter size and computational cost of 1D convolution are much lower than those of 2D convolution, theoretically reducing the convolutional overhead by approximately 67%. This approach not only allows for the precise capture of local fine physiological features, such as the pulse upstroke and rebound waves, along the temporal dimension, but also avoids the redundant computations associated with 2D convolutions. Therefore, this design achieves both model lightweighting and effective feature extraction at the backbone-network level.

First, the preprocessed blood pressure signal is fed into the encoder, where it is downsampled layer by layer through four convolutional blocks (ConvBlocks). The number of signal channels is doubled from 32 to 256 in each layer. Each convolutional block consists of two 1D convolutions, batch normalization, and ReLU activation During encoding, the signal’s temporal dimension is progressively compressed through pooling operations, extracting deep features at different scales. Subsequently, at the bottleneck layer at the network’s bottom, the deep features output by the encoder are further fused and enhanced to improve representational capacity. The decoder restores the signal’s scale layer by layer through upsampling. To compensate for the information loss caused by deep downsampling, features are concatenated with corresponding encoding layers via skip connections. Meanwhile, the four-layer structure balances feature extraction depth and parameter volume, ensuring that long-term blood pressure trends are not missed due to insufficient layers while avoiding overfitting issues often caused by deeper networks. This structure not only extracts local details from the blood pressure signal but also preserves the overall trend of changes, providing fundamental features for subsequent waveform reconstruction.

#### 2.2.3. Blood Pressure Value Output Network

**Figure 3:**
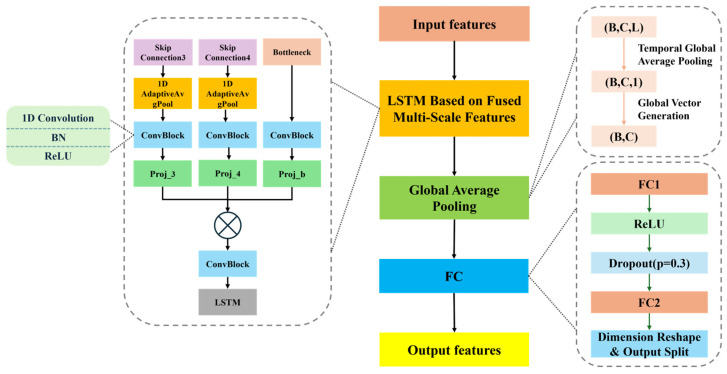
Architecture of blood pressure value output network.

The blood pressure output network consists of an LSTM based on a fused multi-scale features module, a global average pooling module, and a two-layer fully connected prediction head. This architecture leverages the middle-to-deep layer skip connection features and bottleneck layer features from the 1D U-Net encoder to effectively capture multi-scale temporal information from the pulse wave, enabling efficient blood pressure regression prediction. The detailed structure is illustrated in Figure 3.

(a) LSTM Based on Fused Multi-Scale Features

To better handle information from different temporal scales in the 1D U-Net module, this study designs an LSTM temporal modeling module based on multi-scale feature fusion. Different from existing multi-scale feature fusion architectures, the proposed fusion strategy is not a simple stacking of existing network modules. Instead, it is designed as a selective mid-to-deep feature reuse and lightweight fusion strategy tailored for radar-based pulse-wave blood pressure regression.

Traditional multi-scale feature fusion architectures usually enhance feature representation by introducing additional scale-construction pathways or scale-selection mechanisms. The typical logic of FPN-style architectures is to construct a multi-level output feature pyramid: deep features are progressively upsampled through a top-down pathway and fused with the corresponding shallow features via lateral connections, commonly using element-wise addition. This structure promotes layer-wise interaction among features at different levels. But it usually requires additional upsampling operations, lateral 1×1 convolutional transformations for channel alignment, and layer-wise fusion computation. Therefore, it is more suitable for visual tasks, such as object detection and image segmentation, where multi-scale output feature maps are required [25]. Inception-style architectures usually introduce multiple parallel convolutional branches within the same layer. These branches extract feature representations with different receptive fields using convolutional kernels of different sizes or pooling paths, and their outputs are then fused through channel-wise concatenation. Their multi-scale representation capability mainly comes from additionally constructed parallel branches, which increases convolutional computation, the number of parameters, and intermediate feature storage cost [26]. In addition, dynamic weighting fusion methods, such as gating mechanisms or adaptive scale-selection mechanisms, usually require an extra weight-generation module to adaptively assign the importance of features from different scales according to the input features. Although these methods improve fusion flexibility, they also introduce additional parameters, weight computation procedures, and training complexity [27,28].

Different from the above multi-scale fusion methods, the proposed method does not construct an additional top-down feature pyramid, introduce new parallel multi-branch convolutional structures, or employ a dynamic weight-generation module. Instead, it directly reuses the hierarchical temporal features already formed by the encoder of the 1D U-Net during forward propagation. In this design, feature maps at different scales, namely e3, e4, and b, are extracted through skip connections. Here, e1, e2, e3, and e4 represent the output features from different stages of the U-Net encoder through skip connections, while b corresponds to the bottleneck feature at the end of the encoder. These features contain temporal information at different levels. Moreover, shallow features e1 and e2 are not fused in this study because they mainly retain local details and low-level waveform information, which may introduce redundancy and noise into blood pressure regression. In contrast, mid- to deep-layer skip connection features e3 and e4 and the bottleneck feature b provide stronger abstract temporal representations, making them more suitable for subsequent temporal modeling and regression prediction. Therefore, the proposed method is not a naive stacking of U-Net features, but a task-oriented selective multi-scale feature reuse strategy for blood pressure regression.

In terms of the fusion operation, this study adopts a “concatenate first, then project” strategy. Specifically, e3, e4, and b are first concatenated along the channel dimension to preserve the diversity and complementarity of features from different scales. Then, a standard learnable 1×1 convolution is used for channel-level projection and fusion, thereby achieving cross-scale information integration with relatively low computational overhead. The fused single-stream feature is subsequently fed into the LSTM module for unified temporal dependency modeling, allowing the model to capture the dynamic evolution of pulse-wave features over time. Overall, through selective feature reuse and single-stream lightweight fusion, this strategy preserves multi-scale temporal information while reducing additional structural overhead, thereby achieving a balance between feature representation capability and model complexity.

(b) Global Average Pooling

After multi-scale feature fusion and LSTM modeling, the feature tensor still retains temporal information. If directly input into fully connected layers, the high dimensionality would make the model sensitive to the length of the input sequence. Therefore, an effective global aggregation mechanism is required. Therefore, this study uses Global Average Pooling (GAP) [29] to further compress the temporal features output by LSTM into a fixed-dimensional global representation. After completing multi-scale feature fusion and LSTM temporal modeling, the network obtains the temporal hidden representation:

(6) $$F\in R^{B\times C\times L}$$

B represents the batch size, C represents the number of channels, and L represents the temporal dimension length.

The essence of GAP is global average pooling along the temporal dimension, which aggregates the response strength of each channel over the entire duration, providing a global feature description of the waveform. It effectively consolidates global temporal information, preventing the model from overly relying on specific time points. Compared with directly flattening the temporal features output by the LSTM and feeding them into fully connected layers, this study applies GAP along the temporal dimension to independently average each channel. GAP itself introduces no learnable parameters and compresses the feature dimension from C×L to C. If the number of hidden units in the subsequent fully connected layer is H, the number of parameters can be reduced from C×L×H to C×H, thereby substantially reducing the parameter size of the regression head and the overall model complexity. Moreover, GAP demonstrates strong adaptability to sequences of varying lengths, enhancing the model’s robustness. The global average pooling along the temporal dimension is calculated as follows:

(7) $$F_{gap}\left(c\right)=\frac{1}{L}\sum_{t=1}^{L}F(c,t)$$

here, $F_{gap}$ denotes the features after pooling.

(c) Fully Connected Layer

The pooled global features are fed into a two-layer fully connected network for blood pressure regression prediction. The first layer performs high-order feature fusion and dimensional transformation through nonlinear mapping, using the ReLU activation function to enhance the model’s ability to fit complex signal distributions. Additionally, Dropout is introduced to randomly suppress the response of certain neurons, effectively reducing the risk of overfitting and improving the model’s generalization and robustness across radar signals from different individuals. The second layer directly maps the fused features to a two-dimensional output, corresponding to SBP and DBP, achieving continuous blood pressure prediction and providing a simple and efficient prediction output structure for the blood pressure signal regression task [30].

## 3. Experimental Environment and Training Strategy

### 3.1. Two-Stage Hierarchical Training

To reduce the optimization difficulty during the learning process of the neural network, this paper adopts a two-stage hierarchical training framework. The dual-stage hierarchical training framework reduces the model optimization difficulty through step-by-step learning in a shallow-to-deep manner. First, the model learns the basic feature mapping from radar signals to blood pressure waveforms, and then it performs precise regression of blood pressure values. This approach effectively avoids issues such as local optima, chaotic feature learning, and imbalance between waveform and value prediction. Finally, the model achieves a synergistic improvement in both model stability and prediction accuracy.

The first stage is the blood pressure waveform reconstruction phase. The model locks the blood pressure regression head and trains only the 1D U-Net backbone, focusing on learning the feature correspondence between radar time-series signals and blood pressure waveforms. This phase uses the waveform L1 loss function to constrain reconstruction accuracy, continuously reducing the error between the reconstructed waveform and the true waveform through backpropagation, ultimately outputting the blood pressure waveform. The L1 loss function is as follows:

(8) $$L_{wave}=\frac{1}{N}\sum_{i=1}^{N}\left|{{\hat{BP}}_{wave}}_{i}-{{BP}_{wave}}_{i}\right|$$

here, ${{\hat{BP}}_{wave}}_{i}$ and ${{BP}_{wave}}_{i}$ denote the reconstructed and true blood pressure waveform values at the i-th sampling point, respectively. N represents the temporal length of the waveform, namely the total number of sampling points, and i denotes the sampling-point index, ranging from 1 to N.

The second stage is the blood pressure value regression phase. It is executed in two steps based on the pre-trained weights from the first stage. In Sub-stage A, the U-Net backbone parameters are frozen, and only the multi-scale fusion LSTM temporal modeling module and regression head are trained, ensuring that the learned waveform features are preserved. In Sub-stage B, all network parameters are unfreezed, and a smaller learning rate is used for global fine-tuning. A weighted joint loss function is used to simultaneously optimize waveform reconstruction and blood pressure value prediction, ultimately producing outputs for both SBP and DBP. The loss function is as follows:

(9) $$L_{total}=0.3\times L_{wave}+L_{SBP}+L_{DBP}$$

(10) $$L_{SBP}=\frac{1}{N}{\sum_{i=1}^{N}\left({\hat{SBP}}_{i}-{SBP}_{i}\right)}^{2}$$

(11) $$L_{DBP}=\frac{1}{N}{\sum_{i=1}^{N}\left({\hat{DBP}}_{i}-{DBP}_{i}\right)}^{2}$$

here, ${\hat{SBP}}_{i}$ and ${\hat{DBP}}_{i}$ represent the predicted blood pressure values for the i-th sample, while ${SBP}_{i}$ and ${DBP}_{i}$ represent the true blood pressure values for the i-th sample, and N denotes the total number of samples.

### 3.2. Core Training Configuration

#### 3.2.1. Optimizer and Learning Rate Strategy

This paper uses the AdamW optimizer with weight decay, where the weight decay coefficient is fixed at 1 × 10^−5^, addressing the poor weight decay effect of the traditional Adam optimizer and enhancing the model’s generalization. The learning rate scheduling uses the ReduceLROnPlateau strategy. When the validation loss does not decrease for 5 consecutive epochs, the learning rate is automatically reduced to 50% of its original value, avoiding the issue of local optima caused by a fixed learning rate.

The training process of this model consists of three stages: blood pressure waveform reconstruction, freezing the backbone network for training, and global network fine-tuning. Table 1 provides the optimizer parameter configuration for each stage.

#### 3.2.2. Training Epochs and Early Stopping Strategy

The training epochs and early stopping strategy parameters for each stage are as follows. The maximum number of training epochs for Stage 1 is 50, with an early stopping patience value of 15; the maximum number of training epochs for both Sub-stage A and Sub-stage B of Stage 2 is 25, with an early stopping patience value of 20 for both. The maximum number of training epochs represents the preset maximum number of iterations for each training stage, which serves to limit the upper bound of model training and prevent over-training, thereby avoiding waste of computational resources and model overfitting. The early stopping patience value is a key parameter for early stopping regularization. It defines the maximum number of epochs allowed without improvement in the model’s performance on the validation set. Its purpose is to automatically terminate training when the model’s performance reaches saturation and no further optimization is possible, preventing overfitting while improving training efficiency and resource utilization. For example, in Stage 1, if the validation MAE does not improve for 15 consecutive epochs, the training is terminated early without completing the full 50 epochs.

#### 3.2.3. Dataset Configuration

This study uses the publicly available radar vital signs dataset published in *Scientific Data* [31]. The dataset uses a 24 GHz millimeter-wave radar to non-invasively measure the chest of the subjects, capturing non-invasive continuous blood pressure and other physiological signals from 30 healthy subjects across multiple scenarios. The age range of the subjects is 21–61 years, with an average age of 30.7 ± 9.9 years. There are 14 male and 16 female subjects, with an average BMI of 23.2 ± 3.3 kg/m^2^. The radar uses a six-port technology, with a sampling frequency of 2000 Hz for both I/Q channels.

To ensure data quality and minimize motion artifacts, this study uses only the resting-state subset for model training and validation. This subset corresponds to scenarios where the subjects are relaxed, lying down, and breathing calmly. The resting data for each subject lasts no less than 10 min, with a total valid data duration of 19,048.6 s. The data quality has been technically validated using the original dataset. The inter-beat interval correlation coefficient between the radar and electrocardiogram is 96.12%, with no significant motion artifacts or signal loss.

The experimental data come from radar signals and blood pressure synchronized paired data collected from subjects in a resting state. The data samples include continuous radar time-series signals and true blood pressure waveform curves, along with corresponding SBP and DBP annotations. These elements provide a complete supervised learning foundation for the model’s waveform reconstruction and blood pressure regression tasks.

During data preprocessing, both the radar signals and the synchronously acquired blood pressure waveforms were uniformly downsampled to 50 Hz and then segmented into regular 5.12 s windows, with each sample segment containing 256 data points. For each blood pressure waveform segment, the maximum value within the segment was defined as the SBP, whereas the minimum value was defined as the DBP. After Z-score normalization, the processed values are adopted as the supervised labels for the corresponding radar signal segments [21]. Subsequently, all segmented samples were randomly shuffled and divided into training, validation, and test sets at a ratio of 3:1:1, corresponding to 60%, 20%, and 20% of the total samples, respectively. The three subsets were mutually exclusive at the sample-segment level: the training set was used for model parameter learning, the validation set for model selection and hyperparameter tuning, and the test set exclusively for final performance evaluation. The random partitioning procedure described above was controlled using a fixed random seed of 42 to ensure consistent data splits under the same experimental environment and parameter configuration. Therefore, all experimental results reported in this study are based on a single complete training and testing run conducted under the fixed random partitioning scheme described above.

The batch size for model training is set to 16, and the data loading method employs a single-threaded, memory preloading mechanism to avoid frequent disk read/write operations and enhance training stability.

## 4. Results

### 4.1. Blood Pressure Value Prediction

This study evaluates the accuracy and reliability of the model’s blood pressure predictions by quantifying them through three complementary metrics: mean absolute error (MAE) and standard deviation (STD) for systolic and diastolic pressures, alongside the assessment criteria of the British Hypertension Society (BHS). This multi-dimensional approach addresses the magnitude of numerical errors, the dispersion of error distribution, and the conformity with clinical measurement standards.

MAE reflects the average deviation between the predicted and true values. A smaller MAE indicates higher overall predictive accuracy and the absence of systematic bias. The calculation is defined as follows:

(12) $$e_{i}={\hat{y}}_{i}-y_{i}$$

(13) $$\left|e_{i}\right|=\left|{\hat{y}}_{i}-y_{i}\right|$$

(14) $$\bar{e}=\frac{1}{N}\sum_{i=1}^{N}e_{i}$$

(15) $$MAE=\frac{1}{N}\sum_{i=1}^{N}\left|e_{i}\right|$$

Here, $e_{i}$ represents the signed error, $\left|e_{i}\right|$ represents the absolute error, $\bar{e}$ represents the mean error, N denotes the total number of samples, ${\hat{y}}_{i}$ represents the predicted systolic or diastolic pressure for the i-th sample, and $y_{i}$ is the corresponding true value of the i-th sample.

STD reflects the dispersion and stability of the model’s prediction errors. A smaller STD indicates that the prediction errors of individual samples are more concentrated around the MAE, suggesting greater stability of the model’s predictions and the absence of extreme error cases. The calculation is defined as follows:

(16) $$STD=\sqrt{\frac{1}{N}\sum_{i=1}^{N}{\left(\left|e_{i}\right|-\bar{e}\right)}^{2}}$$

The BHS evaluation criteria are a classic clinical standard for assessing the accuracy of blood pressure measurement devices or models. This standard classifies accuracy into four levels: A (optimal), B, C, and D (worst), based on the cumulative percentage of errors between the predicted values and mercury sphygmomanometer reference values falling within ≤5, ≤10, and ≤15 mmHg. It also requires separate ratings for systolic and diastolic pressures. In clinical practice, a device is considered to have reliable measurement accuracy only if it achieves at least a B rating [32]. Table 2 presents the specific evaluation criteria of the BHS.

As shown in Table 3, the model’s testing on the dataset yields mean absolute errors for SBP and DBP of 3.21 ± 4.94 mmHg and 2.25 ± 3.39 mmHg, respectively. As shown in Table 4, the proportion of samples with SBP errors ≤ 5 mmHg, ≤10 mmHg, and ≤15 mmHg in the test set were 81.50%, 96.46%, and 98.43%, respectively. The corresponding proportions for DBP in the test set were 92.32%, 98.43%, and 99.02%, with the results meeting the BHS Class A standard.

**Figure 4:**
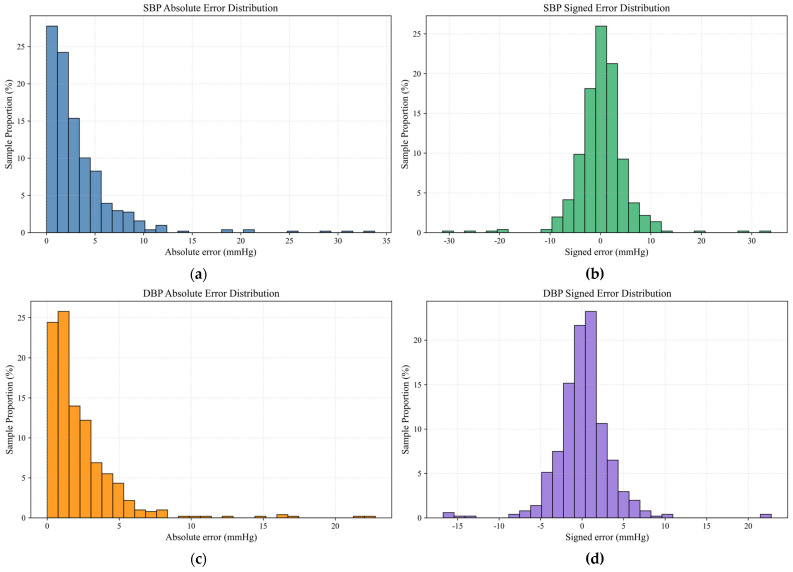
Distribution histograms of SBP and DBP prediction errors: (**a**) SBP Absolute Error Distribution; (**b**) SBP Signed Error Distribution; (**c**) DBP Absolute Error Distribution; (**d**) DBP Signed Error Distribution.

Figure 4 shows the distribution histograms of SBP and DBP prediction errors, evaluating model performance from the perspectives of error magnitude and error distribution. Specifically, absolute error is used to quantify the magnitude of prediction deviation, while signed error is used to characterize the error distribution and possible systematic bias. The absolute error distributions show that the prediction errors for both SBP and DBP are mainly concentrated within small ranges, with very few large-error samples, indicating good overall accuracy. The signed error distributions show approximately symmetric unimodal patterns, with peaks near 0 mmHg, indicating no obvious systematic bias in the model predictions. In comparison, the error distribution of DBP is more concentrated: its absolute errors are mainly distributed within the 0–7.5 mmHg range with a higher peak, while its signed error distribution is narrower with a smaller standard deviation, suggesting better stability and consistency. This is consistent with the quantitative results, where DBP achieves lower MAE and STD than SBP. Overall, the proposed model shows good accuracy and stability in both SBP and DBP prediction tasks, with better performance for DBP, suggesting greater application potential in clinical or daily blood pressure monitoring scenarios.

**Figure 5:**
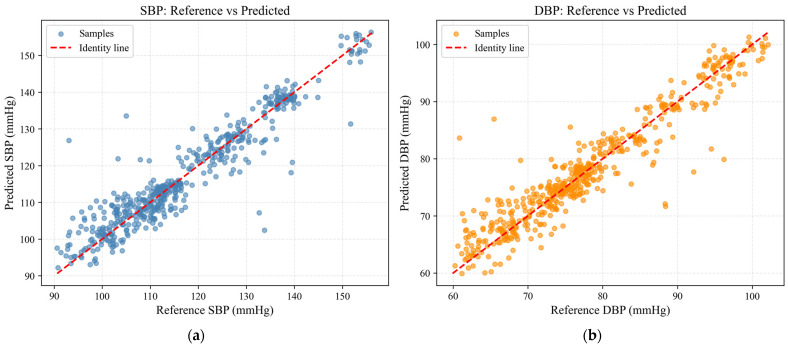
Scatter plot comparison of reference values and predicted values: (**a**) SBP; (**b**) DBP.

Figure 5 shows the scatter plot comparison between the reference values and predicted values for SBP and DBP, with the red dashed line representing the ideal prediction line (y = x). The predicted values for both SBP and DBP exhibit a strong positive linear correlation with the reference values. The scatter points are closely clustered around the ideal line, indicating that the predictions are highly accurate.

**Figure 6:**
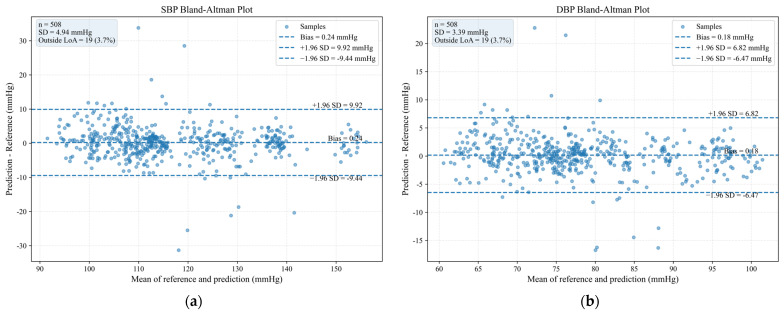
Bland–Altman plot for SBP and DBP: (**a**) SBP; (**b**) DBP.

Figure 6 shows the Bland–Altman plot for SBP and DBP, visually validating the consistency of the model’s blood pressure predictions. In 508 test set samples, the mean bias for SBP was 0.24 mmHg, with an STD of 4.94 mmHg. The 95% limits of agreement were [−9.44, 9.92] mmHg, with 3.7% of the samples falling outside these limits. The scatter points were tightly clustered around the bias line, showing no significant systematic trend with respect to the mean value. The bias for DBP was 0.18 mmHg, with an STD of 3.39 mmHg. The 95% limits of agreement were [−6.47, 6.82] mmHg, with 3.7% of the samples falling outside these limits. The scatter points were similarly stable and closely aligned with the bias line.

In conclusion, the LULMNet can accurately predict the values of SBP and DBP, with minimal deviation from the actual values, demonstrating reliability and stability.

### 4.2. Blood Pressure Waveform Reconstruction

In this study, the waveform reconstruction performance of the model is measured using the Normalized Mean Absolute Error (NMAE) of the entire dataset. NMAE is the arithmetic mean of the MAE for each individual sample waveform, providing a global measure of the model’s waveform reconstruction performance. The lower the NMAE, the stronger the model’s waveform reconstruction capability and the better the overall prediction stability. From a global perspective, the NMAE of this experiment is 0.044, indicating a low error level and validating the model’s effectiveness. The formula for calculating NMAE is as follows:

(17) $${NMAE}_{wave}^{total}=\frac{1}{N}\sum_{i=1}^{N}{MAE}_{wave}^{\left(i\right)}$$

(18) $${MAE}_{wave}^{\left(i\right)}=\frac{1}{C\times T}\sum_{C=1}^{C}\sum_{t=1}^{T}\left|{\hat{y}}_{c,t}^{\left(i\right)}-y_{c,t}^{\left(i\right)}\right|$$

${NMAE}_{wave}^{total}$ represents the normalized mean absolute error of the waveform for the entire dataset, and $MAE_{\mathrm{wave}}^{\left(i\right)}$ represents the normalized mean absolute error of the waveform for the i-th sample. C denotes the number of waveform channels, and T denotes the number of time steps in the waveform. ${\hat{y}}_{\left(c,t\right)}^{\left(i\right)}$ represents the normalized predicted waveform value at the i-th sample, c-th channel, and t-th time step, while $y_{\left(c,t\right)}^{\left(i\right)}$ represents the normalized true waveform value at the same position.

**Figure 7:**
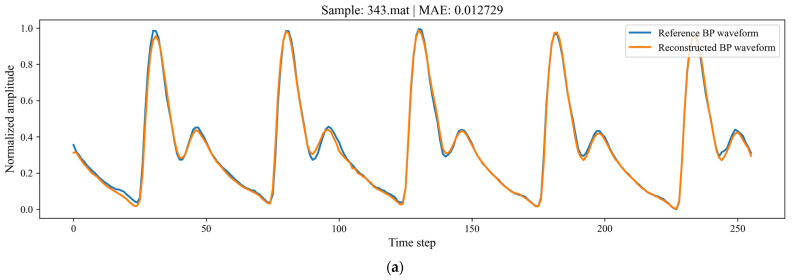

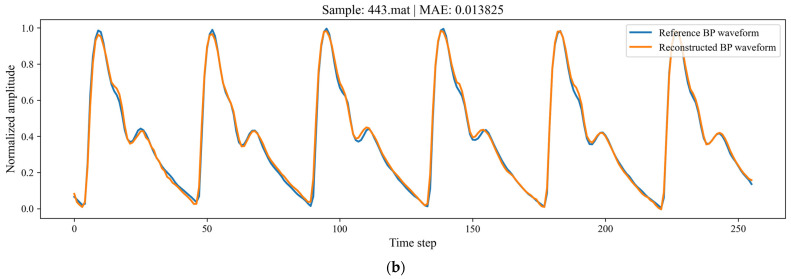
Waveform Reconstruction Graph: (**a**) sample 343; (**b**) sample 443.

Given that average metrics mainly reflect the overall error level and are limited in capturing detailed features such as local morphology, peak and trough positions, and amplitude variations in the reconstructed waveform. This study further selects the reconstruction results of two representative samples for visualization. Figure 7 shows the reconstruction results of the pulse waveforms for samples 343 and 443, with the blue curve representing the true BP waveform and the orange curve representing the model’s reconstructed waveform. From the figure, it can be observed that the model’s reconstructed waveform closely matches the true waveform in terms of overall trend, peak amplitude, trough position, and other key physiological features, with only minor deviations in local details. The MAE for samples 343 and 443 are 0.013 and 0.014, respectively, indicating a small error.

In summary, the model is able to accurately capture the temporal structure and physiological features of the pulse wave, providing a reliable feature foundation for subsequent multi-scale feature extraction and blood pressure regression tasks, thereby validating the effectiveness of the waveform reconstruction module.

### 4.3. Model Interpretability Analysis

To further enhance the interpretability of the deep learning model in blood pressure prediction, this study incorporated saliency map analysis to identify the temporal regions of the blood pressure waveform that contributed most substantially to the prediction of SBP and DBP.

**Figure 8:**
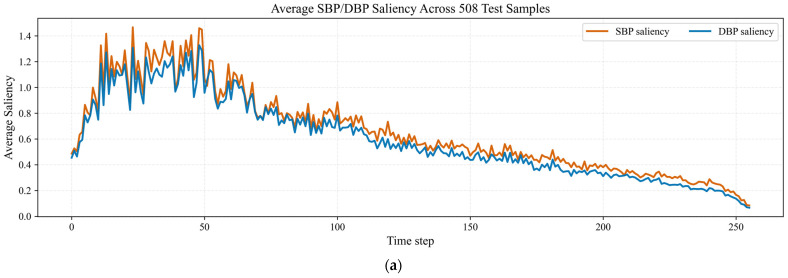

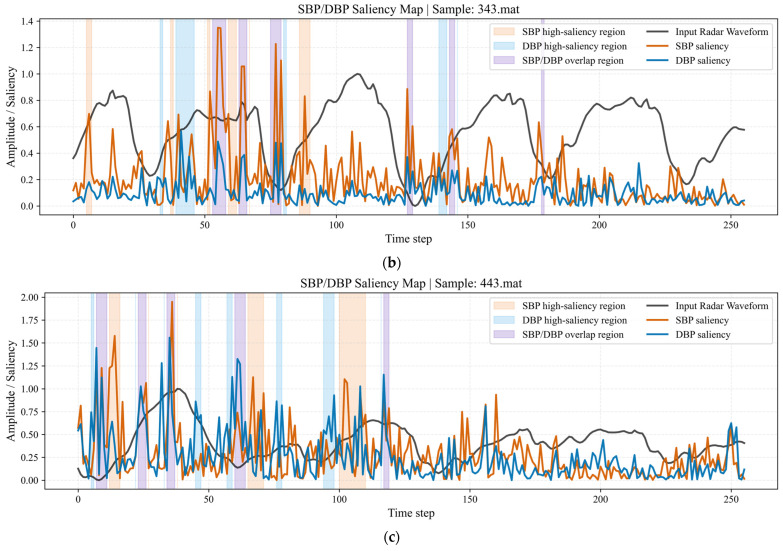
SBP/DBP saliency map analysis. (**a**) Average saliency across 508 test samples. (**b**) Saliency map of sample 343. (**c**) Saliency map of sample 443.

As shown in Figure 8, in the averaged saliency results for the test set, the orange curve denotes the mean saliency for SBP prediction, whereas the blue curve denotes the mean saliency for DBP prediction. These curves are used to compare the temporal contribution patterns of the two blood pressure indices across the overall test samples. In the single-sample saliency map, the black curve represents the input radar waveform, while the orange and blue curves indicate the saliency scores at each time step for SBP and DBP, respectively. The orange shaded regions denote the high-contribution temporal segments ranked within the top 15% for SBP prediction, the blue shaded regions denote those ranked within the top 15% for DBP prediction, and the purple shaded regions indicate the overlap between the high-contribution regions for SBP and DBP.

The results from the representative samples show that the high-contribution regions in samples 343 and 443 are primarily located at positions with pronounced waveform morphological variations, including the rising edges, falling edges, local peaks, and valleys. As shown in Figure 8b, in sample 343, the high-contribution regions for SBP and DBP are relatively dispersed, but they overlap in several critical temporal segments. This indicates that the prediction of both blood pressure indices shares a common dependence on certain local waveform features. As shown in Figure 8c, in sample 443, the high-contribution regions are more concentrated in the first half of the sequence, particularly around the main peak and its adjacent varying regions. This observation is consistent with the overall trend in the averaged saliency map, where the first half of the sequence shows higher contribution levels.

Overall, the model primarily relies on key local temporal information in the first half of the radar waveform for SBP and DBP prediction, rather than assigning uniform or indiscriminate weights to the entire input sequence. SBP and DBP exhibit similar overall attention patterns; however, SBP shows a stronger response to transient local morphological variations. These results indicate that the model’s predictive basis corresponds well to the key temporal morphological features of the radar waveform, thereby enhancing the interpretability of the model in the blood pressure prediction task.

## 5. Discussion

### 5.1. Ablation Experiment

To verify the contribution of each module of the LULMNet model to blood pressure prediction and waveform reconstruction, this study conducts a module combination ablation analysis. During the experiment, the principle of controlling variables is strictly followed, and different combinations of model modules are used, keeping parameters such as the number of training epochs, learning rate, and experimental dataset consistent with the original model. The experiment compares core metrics such as SBP and DBP prediction errors under different configurations, quantitatively analyzing the independent contribution and interactions of each module to model performance, and identifying the key supporting modules that enhance model performance.

Table 5 presents the model composition. To systematically evaluate the contribution of each module to blood pressure waveform reconstruction and regression tasks, four model variants were designed by progressively incorporating additional components. The baseline model (Model 1) consisted only of a 1D U-Net and FC output layer. Model 2 introduced GAP layer to aggregate the extracted features. Model 3 further incorporated a multi-scale feature fusion module based on Model 2. The complete architecture (Model 4) additionally integrated an LSTM layer to capture temporal dependencies in sequential waveform data.

Table 6 shows that with the gradual introduction of the GAP, multi-scale feature extraction, and LSTM modules, the model’s overall performance on SBP and DBP prediction tasks continues to improve. In all tables of this paper, the best result for each metric is marked in bold. Compared to the most basic model (Model 1), the complete model shows a significant reduction in both MAE and STD for the two tasks. Specifically, the MAE for SBP decreases by 29.61%, and the STD decreases by 22.33%; the MAE for DBP decreases by 33.23%, and the STD decreases by 30.82%. This indicates that the introduction of each module effectively enhances the model’s regression capability and prediction stability.

After the introduction of GAP, both SBP and DBP errors show a significant decrease, with reductions of 25.44% (SBP MAE), 9.59% (SBP STD), 14.54% (DBP MAE), and 19.39% (DBP STD), respectively. On the basis of the local waveform features extracted by 1D U-net, GAP further removes non-BP-related waveform variations. These variations arise from respiration, body motion, or baseline drift across different individuals. At the same time, GAP preserves the global energy distribution in the pulse waveform that is most relevant to SBP and DBP. This operation essentially acts as a low-pass information filter. It effectively enhances the model’s sensitivity to BP-relevant signal components. It also reduces the interference caused by inter-individual variability on prediction outcomes.

After changing the model’s single input for blood pressure value output to a multi-scale feature fusion input, the model’s performance further improves. This improvement is particularly noticeable in the error range indicators, where significant gains are observed, such as the accuracy of SBP ≤ 10 mmHg and DBP ≤ 15 mmHg, both of which have further increased by 2.35% and 1.21%, respectively. This indicates that the multi-scale structure is capable of capturing complementary features across different U-Net layers. It addresses the inherent limitations of single-scale convolution operations, which often fail to balance fine local details and global contextual trends. Specifically, convolutional layers at different depths within the U-net naturally have different receptive fields. Small-scale kernels from the middle layers have smaller receptive fields. They excel at capturing rapid local changes in the pulse waveform. Large-scale kernels from the deep layers have larger receptive fields. They excel at capturing slow global trends in the waveform. By fusing these two types of features, the model can leverage both local details and global morphological information. This avoids information loss or feature aliasing caused by a single scale. As a result, the model extracts features more accurately for blood pressure prediction. Ultimately, it improves the joint prediction performance for both SBP and DBP. By these means, the model achieves stronger hierarchical feature representation and better generalization against complex input variations.

After adding LSTM on top of this, the model achieves the overall best results, with a more noticeable improvement in SBP, achieving additional reductions of 12.77% (MAE) and 4.63% (STD), respectively. The core reason is that convolutional modules from the 1D U-Net excel at capturing local morphological characteristics. In contrast, the LSTM leverages its unique gating mechanism to model long-range dependencies within feature sequences, converting static local features extracted by the 1D U-Net into continuous, context-aware dynamic representations. Blood pressure and pulse signals exhibit strong temporal correlation. Single local waveform features cannot fully reflect the variation law of blood pressure, while LSTM can effectively capture the temporal evolution characteristics of pulse waveforms and compensate for the limitations of convolutional networks in global temporal modeling. For blood pressure prediction tasks, this temporal correlation modeling helps the network better understand the overall evolution of the pulse waveform, thus further reducing prediction errors and improving result stability.

Overall, GAP, multi-scale feature extraction, and LSTM enhance the model performance from three perspectives: global feature selection, cross-scale information fusion, and temporal dependency modeling. After their collaboration, the model not only improves the prediction accuracy of SBP and DBP but also enhances the error distribution. This indicates that the constructed network structure can better exploit the valuable information in the pulse wave signal, demonstrating good rationality and effectiveness.

### 5.2. Comparison Experiment

To validate the effectiveness and superiority of the proposed method in radar-based non-invasive blood pressure prediction, this section selects recent mainstream methods based on radar technology and deep learning as comparison benchmarks. A comprehensive comparative analysis is conducted across three dimensions: model complexity, prediction accuracy, and clinical compliance rate. The prediction accuracy and clinical compliance rate data are taken from publicly available results in the corresponding literature, ensuring fairness in the comparison under unified evaluation metrics. Model complexity is estimated based on the components of the network model.

As shown in Table 7, the proposed model outperforms existing methods in key metrics such as mean absolute error and standard deviation for both SBP and DBP. Compared to the current best-performing method by Jiang et al. [21], the proposed method reduces SBP prediction error by approximately 8.02% and the standard deviation by 14.09%; DBP prediction error is reduced by 6.25% and the standard deviation by 5.57%. Compared to other deep learning models, the accuracy improvement is more significant, fully demonstrating the effectiveness of the proposed network structure in radar-based pulse wave blood pressure prediction tasks.

As shown in Table 8, existing methods in the same category generally perform well in larger error ranges (e.g., within a 15 mmHg error range), meeting the basic clinical requirements for non-invasive blood pressure monitoring. However, the prediction accuracy in smaller error ranges (e.g., ≤5 mmHg) still has significant shortcomings, making it difficult to meet the higher precision requirements for clinical applications. In contrast, the core advantage of the proposed method lies in its prediction stability in the smaller error ranges. Under strict evaluation standards such as SBP ≤ 5 mmHg and DBP ≤ 5 mmHg, the compliance rates are 81.50% and 92.32%, respectively, improving by 3.03% and 3.85% compared to Jiang et al. [21]. The improvement over Wang et al. [18], Wang et al. [19], and Qiu et al. [20] is significant. The proposed method effectively addresses the accuracy shortcomings in small error ranges that exist in these previous methods. Meanwhile, within the 15 mm Hg error range, although it does not achieve the best performance among the compared methods, the compliance rates for both SBP and DBP remain high. The LULMNet balance between meeting basic clinical needs and achieving high-precision prediction further demonstrates the clinical utility of the proposed method.

The number of parameters and floating-point operations (FLOPs) are two core metrics for assessing model complexity. A smaller number of parameters means the model occupies less storage space, reducing the requirements for device memory or RAM capacity [33,34]. Lower FLOPs, on the other hand, indicate that the model consumes fewer computational resources, significantly improving inference speed and reducing energy consumption [35].

As shown in Table 9, the proposed LULMNet has only 3.0 M parameters and 0.37 G computational costs. These values are much lower than those of related methods using complex structures, such as ResNet, MultiResUNet, Transformers, multi-head attention, or deformable convolutions. They are only slightly higher than those of the lightweight model proposed by Liu et al. [17]. This demonstrates that the proposed method achieves a favorable lightweight design in terms of both model size and computational cost.

Liu et al. [17] mainly used stacked 1D-CNN translation filters to convert radar phase-difference signals into arterial pulse waves. Deep transfer learning was then used to fine-tune only the top fully connected layer on the target radar data. Therefore, the training and inference structure in the target domain is relatively simple. In contrast, LULMNet constructs a complete two-stage encoder–decoder framework. It not only reconstructs blood pressure waveforms but also performs dual-branch regression for SBP and DBP. Therefore, it has a longer computational path and slightly higher model capacity. However, it also provides stronger waveform representation and pressure estimation capability.

Compared with the ResNet-based methods of Wang et al. [18] and Wang et al. [19], the proposed model avoids the repeated stacking of deep residual blocks. A typical ResNet residual block consists of multiple convolutional layers, batch normalization (BN), ReLU activation, and shortcut connections. As the number of residual blocks increases, the number of convolutional layers, parameters, FLOPs, and intermediate feature storage cost all increase accordingly. In addition, Wang et al. [18] adopted a Transformer to model global temporal dependencies. Wang et al. [19] introduced a multi-head attention mechanism (MHA) after the GRU. Such attention structures require multiple Q/K/V linear projections. They also need to construct an L × L temporal correlation matrix, where L denotes the sequence length. Therefore, their computational cost and memory consumption grow quadratically with the sequence length. In contrast, LULMNet uses a lightweight 1D U-Net to extract multi-scale features. It further employs LSTM and GAP for temporal modeling and global compression. This design reduces the network depth, parameter size, and memory overhead.

Compared with RSD-Net proposed by Qiu et al. [20], the proposed model further reduces the overhead introduced by multi-branch structures and high-complexity attention modules. RSD-Net uses three input streams: CPMV, SCPMV, and RHB. CPMV represents radar chest-wall pulse micro-motion signals. SCPMV is the second-order derivative of CPMV and is used to amplify subtle fluctuations. RHB represents radar heartbeat rhythm signals and provides temporal reference information. In RSD-Net, the SMP-Layer serves as the input backbone layer and contains multi-branch stacked deformable convolutions. Each branch has independent convolutional parameters and follows a separate computational path. In addition to standard convolutional weights, deformable convolutions need to learn sampling offsets and perform dynamic sampling. Therefore, they have higher computational and implementation complexity than standard convolutions. Meanwhile, this method repeatedly introduces channel attention (SE) and MHA. Specifically, SE requires additional channel-wise fully connected mappings, whereas MHA requires Q/K/V projections and an L × L attention matrix. In contrast, LULMNet adopts a single-backbone standard 1D convolutional structure. Therefore, its computational path is more regular, and its deployment complexity is lower.

Compared with the two-stage MultiResUNet method proposed by Jiang et al. [21], the proposed model further simplifies the network architecture while retaining the joint modeling strategy for waveform reconstruction and blood pressure regression. The MultiRes Block in MultiResUNet extracts multi-resolution features through multiple convolutional branches with different scales. Although this design enhances multi-scale representation, it also increases the number of convolutional branches, parameters, and FLOPs. In addition, this method embeds eight-head multi-head self-attention in the encoder. In the second stage, it uses transposed convolution for upsampling, three 1D convolutional layers for dimensionality reduction, and a two-layer LSTM. This results in a relatively long regression path. In contrast, LULMNet obtains multi-scale features using a standard four-layer 1D U-Net. It directly reuses mid- and high-level encoded features for regression. This avoids complex multi-resolution convolutional branches, encoder attention modules, and a deep regression path. As a result, the overall structure is more compact.

In conclusion, even with a significant reduction in model complexity, the proposed method still delivers an overall improvement in core accuracy metrics and clinical compliance rates, showing a clear advantage when the error margin is smaller. Compared to lightweight models with similar model complexity, the proposed model also demonstrates superior overall performance in prediction stability and key clinical metrics.

## 6. Conclusions

This paper primarily addresses the need for non-contact blood pressure measurement using millimeter-wave radar signals and presents a lightweight temporal multi-scale feature fusion network LULMNet. The method employs a two-stage training strategy, with a lightweight 1D U-Net as the backbone, incorporating explicit mid-to-deep layer multi-scale feature fusion, LSTM temporal modeling, and a global feature compression mechanism. This enhances the model’s ability to comprehensively represent local pulse wave details, cross-scale information, and temporal dependencies, enabling accurate prediction of blood pressure values and waveform reconstruction.

The experimental results demonstrate that the proposed method effectively improves the utilization efficiency of radar pulse wave signals, achieving good blood pressure prediction performance and waveform reconstruction while maintaining a lightweight model. This indicates that the model strikes a good balance between prediction accuracy, stability, and computational cost. Compared to existing methods, the proposed approach has certain advantages in network structure design and feature utilization, especially in small error ranges, highlighting that the constructed lightweight multi-scale temporal modeling method can more stably achieve high-precision blood pressure prediction. This validates its effectiveness in non-contact blood pressure prediction tasks.

Despite the progress made in this study, there are still some limitations, and further improvements can be made in the following areas. The current study is primarily based on data from healthy subjects in a resting state, and the model’s generalization ability in more complex populations, diverse scenarios, and real clinical environments still needs further validation. To further evaluate the model’s cross-subject generalization capability, rigorous subject-independent split experiments will be conducted on larger datasets with more participants. Future research will also focus on enhancing interference resistance and achieving integration of hardware and software.

## Figures and Tables

**Table 1:** Optimizer Parameter Configuration for Different Stages.

Training Stage	Optimizer Parameter Range	Initial Learning Rate	Description of Settings
Stage 1	All U-Net encoder–decoder parameters	1 × 10^−3^	Larger learning rate to allow the model to quickly learn the waveform feature mapping
Stage 2 Sub-stage A	Multi-scale feature fusion LSTM and blood pressure regression head parameters	5 × 10^−4^	Moderate learning rate to ensure stable convergence of the new module
Stage 2 Sub-stage B	All model network parameters	1 × 10^−4^	Minimal learning rate for fine-tuning without disrupting pre-trained features

**Table 2:** BHS grading criteria.

	≤5 mmHg	≤10 mmHg	≤15 mmHg
Grade A	60%	85%	95%
Grade B	50%	75%	90%
Grade C	40%	65%	85%
Grade D	fail to reach Grade C criteria		

**Table 3:** MAE and STD of SBP and DBP.

	MAE	STD
SBP	3.21	4.94
DBP	2.25	3.39

**Table 4:** BHS Test Results.

	≤5 mmHg	≤10 mmHg	≤15 mmHg
SBP	81.50%	96.46%	98.43%
DBP	92.32%	98.43%	99.02%

**Table 5:** Model Composition.

Model	1D U-Net	LSTM	GAP	FC	Multi_Scale
1	√			√	
2	√		√	√	
3	√		√	√	√
4	√	√	√	√	√

**Table 6:** Ablation Experiment Results.

Model	SBP					DBP				
	MAE	STD	≤5 mmHg	≤10 mmHg	≤15 mmHg	MAE	STD	≤5 mmHg	≤10 mmHg	≤15 mmHg
1	4.56	6.36	64.76	89.76	97.05	3.37	4.90	79.72	95.08	98.82
2	3.40	5.75	75.39	91.93	97.24	2.88	3.95	84.45	95.89	99.21
3	3.68	5.18	73.62	94.09	98.03	2.55	3.36	86.42	97.05	**99.61**
4	**3.21**	**4.94**	**81.50**	**96.46**	**98.43**	**2.25**	**3.39**	**92.32**	**98.43**	99.02

**Table 7:** Comparison of Prediction Accuracy.

	SBP MAE	SBP STD	DBP MAE	DBP STD
Liu et al. [17]	/	6.12	/	3.78
Wang et al. [18]	5.00	/	3.96	/
Wang et al. [19]	4.90	6.78	3.58	5.13
Qiu et al. [20]	4.86	6.14	4.42	5.50
Jiang et al. [21]	3.49	5.75	2.40	3.59
Our model	**3.21**	**4.94**	**2.25**	**3.39**

**Table 8:** Comparison of Clinical Compliance Rate under BHS Standards.

	SBP ≤ 5 mmHg	SBP ≤ 10 mmHg	SBP ≤ 15 mmHg	DBP ≤ 5 mmHg	DBP ≤ 10 mmHg	DBP ≤ 15 mmHg
Liu et al. [17]	74.7	91.3	97.0	87.0	96.3	**99.7**
Wang et al. [18]	62.74	87.42	95.37	75.49	93.15	97.35
Wang et al. [19]	63.06	87.89	95.67	76.17	93.64	97.86
Qiu et al. [20]	62.84	88.25	**98.91**	61.20	93.17	99.45
Jiang et al. [21]	79.1	93.7	97.8	88.9	98.2	99.6
My model	**81.50**	**96.46**	98.43	**92.32**	**98.43**	99.02

**Table 9:** Comparison of Parameter Count and FLOPs.

	Params	FLOPs
Liu et al. [17]	**1.6 M**	**0.25 G**
Wang et al. [18]	3.6 M	0.69 G
Wang et al. [19]	4.1 M	0.73 G
Qiu et al. [20]	10.7 M	0.93 G
Jiang et al. [21]	19.1 M	1.04 G
My model	3.0 M	0.37 G

## Data Availability

The original data presented in this study are openly available in figshare at https://doi.org/10.6084/m9.figshare.12186516. The dataset contains 24 h of clinically recorded radar vital signs with synchronized reference sensor signals from 30 healthy subjects, including raw radar signals, ECG, impedance cardiogram, continuous blood pressure, and corresponding hemodynamic and autonomic nervous system parameters. All data are stored in .mat format and can be accessed and reused freely.
